# Binding to the Other Side: The AT-Hook DNA-Binding Domain Allows Nuclear Factors to Exploit the DNA Minor Groove

**DOI:** 10.3390/ijms25168863

**Published:** 2024-08-14

**Authors:** Sabrina Battista, Monica Fedele, Luca Secco, Alberto Maria Davide Ingo, Riccardo Sgarra, Guidalberto Manfioletti

**Affiliations:** 1 Institute of Experimental Endocrinology and Oncology “G. Salvatore” (IEOS), National Research Council (CNR), 80131 Naples, Italy; sabrina.battista@cnr.it (S.B.); monica.fedele@cnr.it (M.F.); 2 Department of Life Sciences, University of Trieste, 34127 Trieste, Italy; luca.secco@phd.units.it (L.S.); albertoingo@gmail.com (A.M.D.I.)

**Keywords:** AT-hook, HMGA, PATZ1, ZFAT, LEDGF/p75, MLL1, MeCP2, AKNA, BRG1, EBNA1

## Abstract

The “AT-hook” is a peculiar DNA-binding domain that interacts with DNA in the minor groove in correspondence to AT-rich sequences. This domain has been first described in the HMGA protein family of architectural factors and later in various transcription factors and chromatin proteins, often in association with major groove DNA-binding domains. In this review, using a literature search, we identified about one hundred AT-hook-containing proteins, mainly chromatin proteins and transcription factors. After considering the prototypes of AT-hook-containing proteins, the HMGA family, we review those that have been studied in more detail and that have been involved in various pathologies with a particular focus on cancer. This review shows that the AT-hook is a domain that gives proteins not only the ability to interact with DNA but also with RNA and proteins. This domain can have enzymatic activity and can influence the activity of the major groove DNA-binding domain and chromatin docking modules when present, and its activity can be modulated by post-translational modifications. Future research on the function of AT-hook-containing proteins will allow us to better decipher their function and contribution to the different pathologies and to eventually uncover their mutual influences.

## 1. A General Overview of AT-Hooks

### 1.1. Structural Features of AT-Hooks and Their DNA-Binding Properties

The DNA molecule consists of a ladder-like structure, with the two bearing poles made of phosphate and sugar and the rungs made of hydrophobic nitrogenous base pairs (A/T and G/C) that form a double helix. DNA can adopt different helix conformations (A-DNA, B-DNA, and Z-DNA) as well as different distorted structures, such as cruciform and triplex DNA structures. The most common DNA helix is the B-type, which has two grooves, major and minor, depending on their width. Transcription factors (TFs) usually land on the DNA by making multiple contacts with the DNA via electrostatic interactions, hydrogen bonds, and hydrophobic interactions, which are also favoured by the specific conformations of the DNA molecule itself. The general idea about TFs is that they achieve their specificity through base-specific interactions (hydrogen bonds and hydrophobic contacts) in the major groove. However, some factors can also make contacts with DNA via the minor groove, especially in correspondence of AT-rich DNA sequences, via specific DNA-binding domains (DBD).

A particular feature of the minor groove of AT-rich DNA sequences is that it is highly hydrated, and the water molecules within this structure are fixed in an ice-like organisation, conferring great rigidity to the DNA molecule [1]. When DBDs displace these molecules, the DNA becomes more flexible and can adopt sharply curved structures [1].

**Figure 1:**
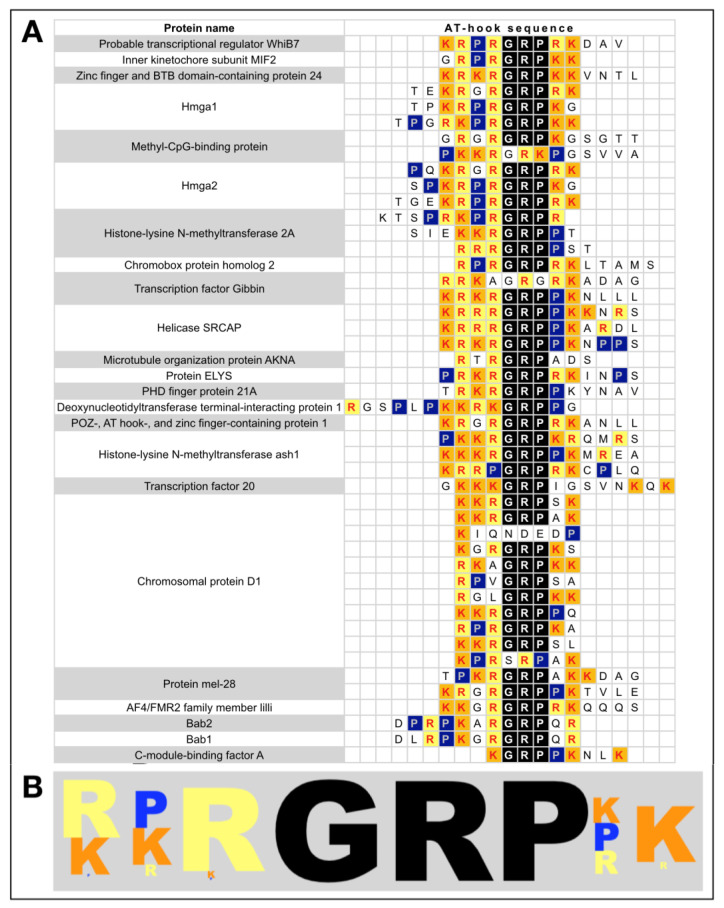
An AT-hook sequence comparison. (**A**) Proteins whose AT-hook sequences are reported in the UniProt Knowledgebase have been included in the table. The sequences of their AT-hook are shown on the right side, and they are aligned, taking as a reference the GRP core (evidenced in black). Arginine (R), Lysine (K), and Proline (P) are evidenced with different colours. Four AT-hook sequences are missing the GRP core. (**B**) A graph depicting the relative abundance of amino acid in the AT-hooks is shown.

The AT-hook DNA-binding domain (DBD), hereafter referred to as “AT-hook”, is a peculiar peptide motif of 10–15 amino acid residues that can fit into the narrow minor groove of DNA and cause bends in the DNA structure. Most AT-hook motifs have a GRP core sequence flanked by basic residues (K or R) and often also by proximal P residues (Figure 1 and Supplementary Table S1 for a comparison of different AT-hooks belonging to different factors). 

However, a recent work has described the existence of non-canonical AT-hook motifs, which are generally defined as AT-hook-like motifs. They are similar in size to canonical AT-hooks but have additional amino acid residues in close proximity of, or within, the GRP core motif [2]. Proteins with non-canonical AT-hook-like domains include AT-hook-containing transcription factor (AKNA), phosphatidylserine receptor, Jmj-domain-containing protein D6 (PSR/JMJD6), capsicum annuum AT-hook-like gene 1 (CaATL1), terminal deoxynucleotidyltransferase-interacting factor 1 (TdIF1), lens epithelium-derived growth factor 75 (LEDGF/p75), and methyl-CpG-binding protein 2 (MeCP2) proteins [3,4,5,6,7,8,9]. More recently, a new non-canonical AT-hook motif, the so-called extended AT-hook (eAT-hook), has been described in the chromatin regulator protein Tip5 (TTF1-interacting protein 5): three times longer than other AT-hooks, the eAT-hook is characterised by a GRP peptide motif flanked by basic amino acid patches located 12 amino acids from it [2].

**Figure 2:**
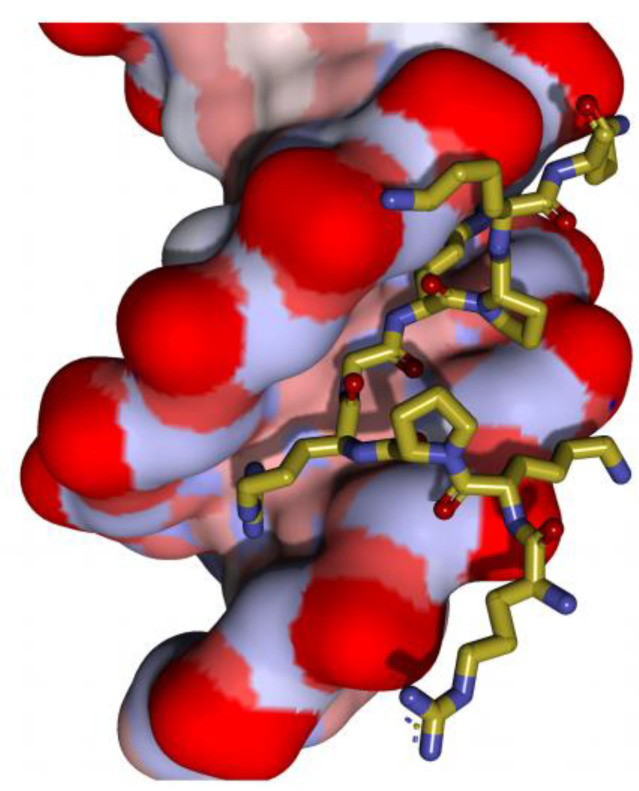
The AT-hook of HMGA1 binding to the minor groove of the DNA sequence. The AT-hook structure with DNA (space filling atoms) evidences the PRGRP core interacting with the inner part of the minor grove of the 5′-CGAATTAATTCG-3′ duplex DNA sequence while flanking basic residues contact the phosphate backbone (picture taken from Fonfría-Subirós et al. [11]).

The interaction of the AT-hooks with DNA has been characterised in detail in the High Mobility Group A (HMGA) protein family, where the AT-hooks have been first described (see Section 2.1. HMGA family) [10,11]. HMGA proteins (HMGA1 and HMGA2) have three AT-hooks. Several studies using NMR and crystallography revealed that the central RGR core fits deeply into the minor groove of the DNA, while the neighbouring basic residues contribute to the binding the DNA backbone through electrostatic and hydrophobic contacts (Figure 2). 

The importance of the RGR core for the DNA-binding of the HMGA AT-hook was also confirmed by a trapped Ion-Mobility Spectrometry–Mass Spectrometry (IMS-MS) approach [12]. It has also been shown that the polar and basic residues in close proximity to the second AT-hook of HMGA1 represent additional elements that confer higher DNA-binding affinity [10] and explain the difference in binding affinity with the other two (the first and the third) AT-hooks, which do not have polar/basic modules. It is plausible to apply these reasonings to AT-hooks of other proteins. For example, structural insights from NMR and crystallography studies on the AT-hook-containing bacterial protein WhiB7 [13], as well as NMR and molecular docking experiments on the AT-hook of BRG1 [14], have confirmed the DNA-binding characteristics observed in HMGA proteins.

It is worth noting that HMGA proteins can also bind distorted DNA structures [15], although no detailed information on the role of AT-hooks in such DNA contacts has been provided.

### 1.2. AT-Hooks in RNA Binding

Although AT-hooks have always been referred to as DNA-binding domains, they can also bind to RNA molecules. It has been demonstrated that the AT-hooks of HMGA1 play a crucial role in recruiting the origin of replication complex (ORC) to the Epstein–Barr virus origin of plasmid replication (OriP). This recruitment is due to a specific interaction between AT-hooks and structured G-rich RNA molecules [16]. It was also shown that the first AT-hook of HMGA1 is able to bind to 7SK RNA, an RNA molecule that forms an snRNP involved in the modulation of RNA pol II activity by masking positive transcription elongation factor-b (P-TEFb) [17]. Furthermore, the first AT-hook also binds to the nascent transcript of the HIV-1 genome, i.e., the transactivating response element (HIV-1 TAR) [18]. In all these cases, the RNA molecules form a stem–loop structure. Interestingly, the latter two studies have clearly shown that only the first AT-hook of HMGA1 is responsible for binding to the RNA molecule, while the second and third are not involved.

Another example of the RNA-binding activity of HMGA1 is provided by the binding to the mRNA of presenilin-2 and oestrogen receptor alpha, whereby HMGA1 would affect their splicing, though not necessarily through its AT-hooks [19,20,21].

Interestingly, the non-canonical eAT-hook binds with higher affinity to RNA than to DNA and is required for the nucleocytoplasmic shuttling of ribonucleoprotein particles [2]. 

### 1.3. AT-Hooks and Their Involvement in the Process of Base Excision Repair (BER)

DNA is exposed to a plethora of endogenous and exogenous genotoxic agents. When DNA bases are damaged or mutated, they must be removed and replaced by the information present in the undamaged complementary DNA filament. Base removal is carried out by specialised DNA glycosylases that recognise the various damages/mutations, remove the damaged bases, and thus create apurinic/apyrimidinic (AP) sites. The repair process requires the activity of a DNA polymerase, which must have free access to the template strand. For this reason, the DNA must be cleaved by two specific lyases, the AP and dRP lyase, in order to generate suitable 3′-OH and 5′-P ends for DNA polymerase [22].

In a paper by Droge’s group, it was shown that the basic residues within the AT-hooks of HMGA exhibit AP and 5′-deoxyribosyl phosphate (dRP) lyase activities. The lysine and arginine residues present within the AT-hooks act as nucleophiles capable of attacking AP sites. It was also shown that a high expression of HMGA proteins plays a protective role in such DNA lesions, as this lyase activity is essential for their efficient removal [23]. The authors also emphasised the protective role of HMGA proteins against anticancer chemotherapeutic agents with DNA-damaging effects.

### 1.4. Post-Translational Modifications Affecting the Properties of AT-Hooks

To fully understand the mechanism of AT-hooks binding to DNA, it is also crucial to decipher the modulating effect of post-translational modifications (PTMs). The absence of the N-terminal and C-terminal extensions of AT-hooks has a strong influence on the DNA-binding affinity of AT-hooks. Short AT-hook peptides (5-7-11 residues long centred on the PRGRP core) have low DNA-binding affinity [24], implying that the additional N- and C-terminal residues contribute significantly to enhancing DNA-binding affinity. It is important to note that serine and threonine residues are often present within these proximal sites and are frequently exposed to phosphorylation events, which are widely described as responsible for reducing DNA-binding affinity [25]. This is consistent with the fact that phosphorylation adds two negative charges, exerting repulsive forces with respect to the sugar–phosphate backbone. There are several papers describing the influence of phosphorylation on the DNA-binding activity of AT-hook-containing proteins, particularly in relation to HMGA proteins [26,27,28,29,30,31]. While these effects have been described in studies using naked DNA, there is a very interesting study that investigated the effects of phosphorylation in close proximity of AT-hooks in vivo [32]. It has been shown that such phosphorylation reduces the mobility of HMGA1 in heterochromatin and on the chromosomes. This means that phosphorylated HMGA1 is bound to chromatin with a higher affinity. HMGA1 can bind to nucleosomes, and therefore, the positive contribution of phosphorylation to chromatin interaction could be explained by the interaction with the positively charged histone tails protruding from the nucleosome core. It is also important to emphasise that the protein/protein interaction domain of HMGA proteins has been shown to contain the second AT-hook [33,34], and therefore, any PTM in the vicinity of this domain could have a profound effect on protein/protein interactions as well.

Another PTM-influencing protein charge is lysine acetylation. The acetylation of HMGA1 at the level of K65, which is located in close proximity of the second AT-hook core (…RGRPKGSKac..), is involved in the destabilisation of the IFN-beta enhanceosome [35]. Importantly, thermodynamic studies on the effect of K65 acetylation on DNA-binding affinity have shown that this modification alone is not sufficient to abolish DNA-binding. Therefore, the mechanism responsible for the disassembly of the enhanceosome may involve other factors beyond a simple reduction in DNA-binding affinity [36].

The third PTM that is important for the functional modulation of the AT-hook is arginine methylation. In this context, the AT-hooks of HMGA proteins have been shown to undergo methylation at the level of the central arginine residues (R23, R25, R57, R59, R83, and R85) [37,38,39,40,41,42,43,44,45,46]. Although the effects of this methylation on DNA-binding affinity have not been experimentally investigated yet, it has been hypothesised that the introduction of a methyl group at the arginine guanidinium group may have a destabilising effect.

It is noteworthy that all the aforementioned PTMs could influence not only the canonical DNA-binding activity of AT-hook-containing proteins, but also the various other activities attributed to these specific domains. For example, lysine acetylation might affect the contribution of AT-hooks to lyase activity.

## 2. Proteins Containing AT-Hooks: An Overview

**Figure 3:**
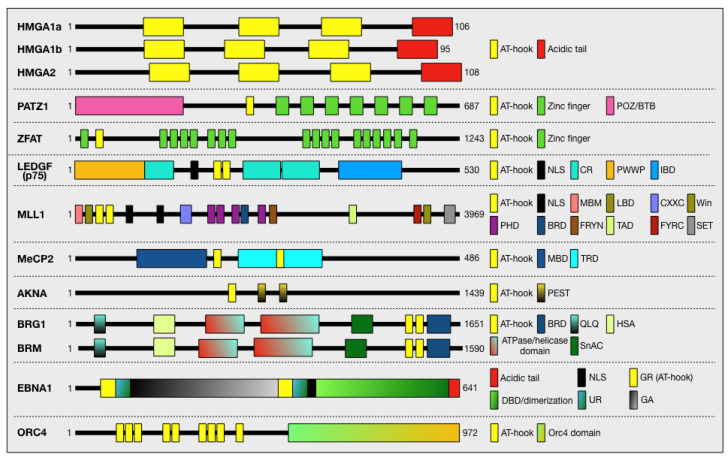
The domain organisation of the AT-hook-containing proteins. A scheme of all the AT-hook-containing proteins described in the text is shown. The domain organisation of HMGA1a, HMGA1b, HMGA2, PATZ1, ZFAT, LEDGF/p75, MLL1, MeCP2, AKNA, BRG1, BRM, EBNA1, and spORC4 is shown. For each protein, the amino acid number is indicated. Proteins and domains are not in scale. POZ/BTB: POxvirus and Zinc finger/Broad-complex, Tramtrack and Bric a brac; NLS: nuclear localisation signal; CR: charged region; IBD: integrase-binding domain; PWWP: proline (P), tryptophan (W), tryptophan (W), proline (P) domain; MBM: menin-binding motif; LBD: LEDGF-binding domain; CxxC: CxxC domain; PHD: plant homology domain; BRD: bromodomain; FYRN: F/Y-rich N terminus domain; TAD: transactivation domain; FYRC: F/Y-rich C terminus domain; Win: WDR5 interaction motif; SET: Drosophila proteins Su(var)3-9, Enhancer-of-zeste and Trithorax domain; MBD: methyl-CpG-binding domain; TRD: transcriptional repression domain; PEST: proline [P]-, glutamic acid [E]-, serine [S]-, and threonine [T]-rich domain; QLQ: glutamin (Q), leucin (L), glutamin (Q) motif; HSA: helicase/SANT-associated domain; SnAC: Snf2-ATP coupling domain; GR: glycine (G)- and arginine (R)-rich domain; DBD: DNA-binding domain; UR: unique region; GA: glycine (G) and alanine (A) repeat region.

In a PubMed search with the keyword “AT-hook”, we found 861 entries, among which we searched for the various proteins associated with this term. In total, about one hundred proteins were found, which are listed in Supplementary Table S1. We found that the function of the AT-hook domain(s) has been characterised for only a subset of proteins, while for the others, only the presence of this domain in their primary structure has been indicated. In the next chapter, we will provide a detailed description of a selected number of proteins focussing on those involved in cancer and/or that have been investigated in more detail, giving functional evidence for the role of the AT-hook domain in their activity. Figure 3 provides a schematic overview of the selected proteins and highlights their functional domains. Proteins belonging to the AT-hook motif nuclear localised (AHL) family of proteins have not been listed in Figure 1 and Supplementary Table S1 and have not been described because they have been recently reviewed [47].

### 2.1. HMGA Family

#### 2.1.1. General Characteristics

The High Mobility Group (HMG) is the second most abundant group of nuclear proteins after histones. The HMG refers to the high mobility of these non-histone proteins in acetic acid–urea gel electrophoresis, the preferred method for analysing histone proteins [48]. Subsequent studies suggested that these nuclear proteins can be categorised into three families according to the characteristic functional domain used to bind DNA: HMGB (HMG box), HMGN (nucleosomal binding domain), and HMGA (AT-hook). The HMGA family is characterised by the presence of three highly conserved DNA-binding domains called AT-hooks. The family consists of three proteins, including HMGA1a and HMGA1b, which are the result of the alternative splicing of the HMGA1 gene, and the highly related HMGA2, which is the product of a similar gene [49]. HMGA are small molecules (about 100 aa residues) rich in basic amino acids, characterised by, in addition to the DNA-binding domains, a highly negatively charged C-terminal tail involved in the modulation of HMGA protein activities (Figure 3) [50,51,52]. HMGA proteins are considered prototypes of the intrinsically disordered (ID) protein category. These proteins are characterised by the lack of a defined secondary and tertiary structure (when free in solution), which gives them a high flexibility responsible for their ability to interact with a large number of molecular partners [34,53,54]. In addition, the presence of multiple sites subject to various constitutive and inducible PTMs is responsible for the fine modulation of their interaction network [53].

All three HMGA proteins show high homology in their three positively charged DNA-binding domains (AT-hooks) and have a highly negatively charged C-terminal tail (Figure 3). The different spacing of the AT-hooks in the three protein forms provides a modular system able to interact with AT-rich DNA regions at different distances from each other. The regions outside their DNA-binding domains differentiate the three proteins both in terms of AT-hook spacing and amino acid sequence [51]. The relatively short dimension, ID status, and “relaxed” ability to specifically bind AT-rich sequences in the DNA minor groove give these factors the ability to interact with a large number of partners and form highly interconnected nodes in the chromatin network [50,53].

#### 2.1.2. AT-Hooks and Their Role in Determining DNA-Binding and Protein Activity

The term AT-hook was first proposed by Reeves when studying the binding of HMGA to DNA [55] and defined as a short peptide with the consensus sequence TPKRPRGRPKK, that is able to bind the narrow minor groove of AT-rich DNA segments, and that is structurally similar to the minor groove binding molecules distamycin A, netropsin, and Hoechst 33258. Nuclear magnetic resonance and crystal structure studies with single AT-hooks and recombinant HMGA proteins containing more than one AT-hook revealed the importance of a central RGR motif within the AT-hook and the ability to bend the DNA and widen the minor groove [10,11]. The binding of HMGA to DNA has been investigated in vitro using PCR-based methods (SELEX) [56] and in vivo using ChIP-Seq in combination with a Hi-C genome analysis [57,58,59]. The combination of these approaches revealed the preferential binding of HMGA to AT-rich sequences across multiple genes involved in regulating a transcriptional network controlling proliferation and cell fate. Additionally, HMGA proteins were found to bind to AT-rich sequences at the level of heterochromatin regions associated with the nuclear lamina, which is crucial for the organising of 3D chromatin structure. This evidence supports an architectural role for HMGA in both the heterochromatin regions and the regulatory regions of several genes. 

RNA-Seq experiments suggest a relevant role for HMGA in regulating a transcriptional network by organising complexes at the level of promoters and enhancers—by binding to both DNA and nuclear transcriptional regulators [60,61]. The involvement of HMGA1 in the formation of the interferon-β gene (IFNB) enhanceosome, proposed by Thanos [62,63] and further supported by additional experimental evidence [64,65], remains a paradigm in the mode of action of HMGA. Thus, HMGA proteins do not have intrinsic transcriptional activity per se. Instead, through their AT-hooks and their capability to interact with multiple partners, they modify DNA and chromatin structure. This allows them to engage with the transcriptional machinery, thereby exerting both positive and negative regulatory effects on the transcription of multiple genes. 

#### 2.1.3. Biological Functions

The ability of HMGA proteins to be involved in local and global changes in chromatin structure is the basis for their contribution to various physiological and pathological processes. The involvement of HMGA in embryonic development was first suggested by the discovery that the pygmy phenotype—mice with a reduced overall size—is related to the deletion of the Hmga2 gene in their genome [66]. Later, the single KO of the Hmga2 gene and the double KO of both Hmga1 and Hmga2 genes showed defects on body size, which result from the decreased proliferation and altered differentiation of various tissues, especially fat and skeletal muscle [67]. The role of HMGA proteins in stem cell maintenance has been demonstrated by their ability to regulate the proliferation and differentiation of various tissue stem-progenitor cells, including mesenchymal, haematopoietic, muscle, and neural stem cells [68,69,70]. Finally, the involvement of HMGA in cellular senescence is supported by the implication of HMGA2 in the formation of senescence-associated heterochromatic foci that accumulate during this process [71].

Given their involvement in diverse biological processes, it is not surprising that alterations in their structure and/or expression can lead to various pathologies, most notably cancer, but also other diseases such as diabetes and other syndromes [72,73,74,75,76].

Since their discovery, HMGA proteins have been associated with cancer, and indeed, their involvement in the onset and development of cancer has been largely demonstrated. In particular, the overexpression of HMGA proteins is a prevalent feature in cancer development, and their causal role in nearly all cancer hallmarks is widely recognised. Both genes are overexpressed in a variety of cancers, and several approaches have shown that they influence the transcription of several genes that are critical for cancer development and progression [60,72,77,78]. HMGA gene rearrangements—especially HMGA2—due to chromosomal translocations are a feature of human benign tumours of mesenchymal origin [79,80]. In most cases, the breakpoint is located in the third intron of the HMGA2 gene, leading to deregulation of its expression, its truncation, or, more commonly, the formation of fusion genes encoding chimeric transcripts containing the first three exons of HMGA2 encoding the three AT-hooks and ectopic sequences of other genes. This has led to speculation that the ectopic sequence (or the lack of a C-terminal tail of HMGA) may confer novel functions to the AT-hooks, leading to the misregulation of HMGA target genes.

In summary, the ability of these small proteins to bind and modify DNA structure enables them to shape chromatin plasticity and regulate gene expression, thereby impacting the onset and progression of several diseases.

### 2.2. POZ/BTB and AT-Hook-Containing Zinc Finger Protein 1 (PATZ1) 

PATZ1, also known as MAZ-related factor (MAZR), zinc finger protein 278 (ZNF278), or zinc finger and BTB protein 19 (ZBTB19), is a versatile transcription factor, that has been known since 2000 [81,82,83] and involved in various biological processes and human diseases, including embryogenesis [84], T-cell development [85], cell senescence [86], DNA damage response [87], the maintenance of neural and embryonic stem cells [88,89,90], metabolism [90], cell reprogramming [91], cell cycle [92,93], apoptosis [94,95], immunodeficiency virus type 1 (HIV-1) infection [96], and a growing list of human cancers, for which PATZ1 has been proposed as a diagnostic and prognostic biomarker [97,98,99,100,101,102,103]. Patz1-KO mice exhibit developmental and neoplastic diseases [94,102], and the dysregulation of PATZ1 has indeed been implicated in cancer progression and other pathological conditions in mice and humans [100,102,104].

The PATZ1 gene generates four alternatively spliced transcripts encoding four protein isoforms, all of which have a POZ/BTB domain at the N-terminus, four to seven C2H2 zinc fingers at the C-terminus, and an AT-hook domain in the middle (Figure 3) [102]. The POZ/BTB domain is found in over 300 human proteins and is involved in mediating protein/protein interactions with repressors, co-repressors, or other proteins carrying POZ/BTB domains, including PATZ1 itself and its isoforms [82,105,106,107]. The AT-hook and the C2H2 zinc fingers are mainly DNA-binding domains that recognise AT-rich sequences and GC-rich specific responsive elements, respectively [108], suggesting distinct roles for PATZ1 as both a chromatin architectural protein and a transcription factor. As a transcription factor, it can either activate or repress transcription, establishing numerous interactions with other proteins, including histone deacetylases and co-repressors N-CoR and SMRT for transcriptional repression and other more typical transcription factors for transcriptional activation/repression [102]. In fact, PATZ1 is not a typical transcription factor, as it lacks an activation domain but regulates transcription by recruiting classical transcription factors. For this reason, its activity is dependent on the cellular context and the expressed proteins [94]. It can either activate or repress the same gene in different cell lines, as has been described for the regulation of c-MYC, BAX, CDKN1A, and MDM2 [81,82,94]. Consequently, PATZ1 can either activate or inhibit apoptosis, depending on the cellular context and, in particular, the expression of a wild-type p53 [94]. Therefore, targeting PATZ1 in advanced stages of carcinogenesis, where p53 function is mostly lost, could be an effective adjuvant therapy to enhance chemotherapeutic effects. PATZ1 and p53 frequently share occupancy of many promoter regions and jointly regulate the expression of multiple genes. In the presence of PATZ1, the transcriptional activity of p53 is enhanced, as demonstrated with CDKN1A, BAX, and MDM2 in HEK293 cells [94] and CDKN1B in HepG2 liver cancer cells [93]. Conversely, in HCT116 colon cancer cells, PATZ1 can inhibit p53’s transcriptional activity, as evidenced by its interference with p53’s DNA-binding or competition for identical consensus regions observed with CDKN1A and BCC3 [87]. Indeed, PATZ1 is required for the proper activation of p53 on MDM2 and CDKN1A promoters [94]. On the other hand, p53 is required for the regulation of CDKN1B by PATZ1 in liver cancer cells [93], while it inhibits the binding of PATZ1 to its specific consensus site in the Cd8 gene [87]. In certain instances, PATZ1 enhances the p53-driven gene expression programme and can independently activate or downregulate the same p53-dependent genes when p53 is absent, effectively substituting for its role [109]. Conversely, PATZ1 can also toggle the transcriptional activity of certain transcription factors, such as Bach 2, from repressive to activating [82]. Additionally, it can dampen the transcriptional activity of other transcription factors, as exemplified by its interaction with RNF4 at the androgen receptor promoter [106] or with EGR1, SP1, and KLF6 at the TGFB1 promoter [110]. In a study using chromatin immunoprecipitation (ChIP) followed by DNA sequencing (ChIP-Seq) on mouse embryonic stem cells, a total of 4587 putative PATZ1 binding sites were annotated, of which 50% were within introns, while only 7% and 3% were within the proximal and distal promoter, respectively [89]. The binding sites consisted of two distinct motifs: a 21-nucleotide sequence with a G-rich region similar to the previously described responsive element [82], which was hypothesised to be the binding site for the zinc fingers, and a 41-nucleotide sequence harbouring a poly-A stretch, which was proposed to be the target site for the minor-groove-binding AT-hook domain [89]. A recent ChIP-Seq analysis in HepG2 liver cancer cells, identified 3683 PATZ1 binding sites across 3005 distinct genes. Of these, 35% were in gene promoters within 1 kb from the TSS, 32% were in introns, 0.54% were in the 3′-UTR, and 3.23% were in the 5′-UTR [93]. The PATZ1 binding sites consist of three responsive elements: one of 24 nucleotides is GC-rich, as previously described [82,89]; the other two of 29 and 21 nucleotides are G-rich, in agreement with the DNA-binding affinity of the zinc fingers [93,111]. In this study, it seems that PATZ1 binding sites do not include AT-rich sequences. This observation suggests that the function of the PATZ1’s AT-hook might be limited to mediating protein/protein interactions, as demonstrated in its interaction with RNF4 [81]. On the other hand, a negatively charged region between zinc fingers motifs 6 and 7 in the C-terminal domain of PATZ1, is involved in the interaction with p53 [87].

In summary, PATZ1 plays a crucial role in gene regulation and key cellular processes, such as cell differentiation, proliferation, and apoptosis, and its dysregulation has been implicated in several diseases. It mainly acts as a transcriptional repressor by interacting with various proteins and DNA elements and exerting its effect on gene expression networks involved in development and disease. 

### 2.3. Zinc Finger and AT-Hook Domain Containing (ZFAT)

The ZFAT gene encodes a protein containing one AT-hook and 18 C2H2-type zinc finger domains (Figure 3). It was originally identified as a candidate susceptibility gene for an autoimmune thyroid disease [112]. It also plays a role in development, primitive haematopoiesis, angiogenesis, the immune response, and various common diseases, such as multiple sclerosis, hypertension, and cancer [113]. ZFAT is a transcriptional regulator recently found to be involved in the control of the centromeric non-coding RNA (ncRNA) transcription in human and mouse cells. By binding to a specific centromeric region, in the presence of centromere protein B (CENP-B) and death domain-associated protein (DAXX), ZFAT recruits the histone acetyltransferase KAT2B. This causes the acetylation at lysine 8 of histone H4 (H4K8ac), resulting in the recruitment of the bromodomain-containing protein BRD4 to the centromeres and leading to the transcription of ncRNA [114,115,116]. Similar to PATZ1, ZFAT is also a crucial regulator of both thymocyte differentiation and peripheral T-cell homeostasis, as it is involved in the development of CD4(+) CD8(+) thymocytes and the definition of peripheral T-cell number through the expression of interleukin-7 receptor-alpha [117,118]. In addition, it is crucial for the maintenance and differentiation of adipocytes [119], the development of erythroid cells in the foetal liver [120], and haematopoietic differentiation in blood islets through the direct regulation of transcription factors [121]. In mouse embryonic fibroblasts and the lymphoblastic leukaemia cell line MOLT-4, ZFAT acts as a pro-survival factor by inducing BCL-2 and IL6st-mediated signalling pathways [122]. Interestingly, ZFAT, like HMGA2, is associated with human height and intelligence [123,124,125,126]. A ChIP-seq analysis revealed that ZFAT predominantly binds to an 8 bp nucleotide sequence GAA(T/A)(C/G)TGC region around the TSS. In addition, about half of the ZFAT-binding sites were characterised by histone H3 acetylation at lysine 9 and lysine 27 (H3K9ac/K27ac) [127]. 

In summary, ZFAT functions as a transcription factor involved in various cellular processes. It regulates gene expression related to immune responses, apoptosis, and cell cycle progression. ZFAT also plays a role in the maintenance of immune homeostasis and is involved in autoimmune diseases. Its interactions with other proteins and DNA elements contribute to its multifaceted role in cellular function.

### 2.4. Lens Epithelium-Derived Growth Factor (LEDGF/p75)

The PC4- and SF2-interacting protein 1 (Psip1) encodes two protein isoforms with molecular masses of 52 kDa and 75 kDa. The latter is also known as LEDGF/p75 (Figure 3), as it was originally described as a growth factor produced by lens epithelial cells and has been reported to play a role in lens epithelial cell survival [128]. They are chromatin-associated proteins that have been implicated as co-activators in transcriptional regulation, mRNA splicing, HIV integration, and cell survival [129]. The two isoforms share a PWWP domain, which is typical of chromatin-associated proteins, and two AT-hooks. In addition, the p75 isoform has an integrase-binding domain (IBD) that tethers HIV-1 enzyme integrase (IN) to host chromosomes, preventing IN degradation [130], and generally plays an important role in tethering protein complexes to chromatin [131]. Most KO mice lacking the Psip1 gene die perinatally, with the rare survivors displaying skeletal anomalies reminiscent of homeotic changes [129]. This implies that Psip1 might play a role in regulating Hox gene expression or act downstream of Hox function. Later on, Yokoyama et al. confirmed in a subsequent study that LEDGF/p75 participates in the MLL/menin-mediated control of Hox genes transcription [132].

LEDGF/p75 is a member of the hepatoma-derived growth factor (HDGF) protein family. Structurally, it is a 530 amino acid protein and consists of the following domains: an N-terminal PWWP domain (residues 1–93) with a conserved Pro-Trp-Trp-Pro sequence, known to interact with methylated lysine 36 of histone H3 [131] and found in various chromatin-associated proteins [133]; a nuclear localisation signal (NLS) and two AT-hook-like motifs enabling non-specific DNA-binding (residues 146–197); and a C-terminal integrase-binding domain (IBD, residues 347–429), also present in another HDGF family member, HDGF-related protein 2 (HRP-3) (Figure 3). LEDGF forms a complex with HRP-3 to relieve the nucleosome-induced barrier to transcription in differentiated cells [134]. Through C-terminal interaction with the lentiviral IN enzyme and N-terminal binding to chromatin via the PWWP domain, LEDGF/p75 tethers the viral IN enzyme to host cell chromatin to enable its integration, as occurs during HIV-1 integration [135,136]. The suggested model is that LEDGF functions as a molecular adaptor for tethering HIV-1 IN within the nucleoprotein complex called the pre-integration complex (PIC), thereby promoting the integration process [137]. Llano et al. have shown that in addition to the PWWP domain and its downstream charged region CR1, a tandem pair of AT-hooks in combination with at least one of the two identified downstream charged regions (CR2 or CR3) is required for this activity [135]. Specifically, a tripartite DNA-binding element, consisting of the NLS and the two AT-hooks, mediates the association of LEDGF/p75 with chromatin in vivo [7]. Recently, using an in vitro model, McNeely et al. confirmed that the AT-hooks and the NLS are both necessary. Specifically, the PWWP domain alone was unable to bind DNA, but it did so when NLS and AT-hooks were also present, likely mediating the binding to naked DNA. Indeed, LEDGF/p75 binds to IN via the IBD, and the NLS and AT-hook-like domains bind the LTR oligo, tightening the complex of IN and DNA [138]. In fact, the PWWP domain is not essential for the HIV-1 cofactor activity of LEDGF [139]. It is likely that the in vivo situation is a combined effect of AT-hooks and NLS-mediated DNA-binding and PWWR-mediated chromatin tethering, resulting in stronger chromatin tethering of IN [138]. In addition, a short peptide spanning residues 178 to 197 of LEDGF/p75 and comprising its AT-hook DNA-binding elements improves the solubility of protein and nucleoprotein complexes of HIV-1 IN with viral DNA ends, sufficient for maximal stimulation of DNA integration [140]. On the other hand, HIV-1 IN enhances LEDGF/p75 chromatin binding during HIV-1 infection, thereby overcoming other LEDGF/p75 interactions with cellular proteins, including the menin/MLL complex [139].

In summary, LEDGF/p75 is a multifunctional protein involved in chromatin organisation and gene regulation. It serves as a transcriptional co-activator, anchoring HIV-1 integrase to host chromatin, thereby aiding viral integration into the genome. Additionally, LEDGF/p75 contributes to DNA repair, the regulation of splicing, and the response to cellular stress. The dysregulation of LEDGF/p75 influences HIV-1 replication, cancer development, and other pathological conditions.

### 2.5. AT-Hook Transcription Factor (AKNA) 

AKNA is an AT-hook transcription factor, with two AT-hook domains at the N- and C-termini: polymorphisms in one of them have been identified as risk factors for cervical cancer (Figure 3) [141]. The gene, located at a fragile site on chromosome 9, encodes 9 different tissue-specific transcripts and can undergo loss-of-function mutations responsible for inflammatory diseases such as knee osteoarthritis (KOA), primary ciliary dyskinesia (PCD) [142], and cancer [142]. The expression of AKNA is finely regulated both transcriptionally by the PKA/CREB and NF-κB pathways [143] and post-transcriptionally by p53 and the proteasomal machinery [144]. Similar to what occurs to HMGA1, the AKNA protein turnover is mediated by the presence of three proteolytic PEST domains [3,145], and it recognises AT-rich motifs in a variety of genes that control multiple cellular processes [146]. AKNA is particularly expressed in germinal centres and immune cells, where it regulates T-cell activation by modulating the expression of cytokines and costimulatory molecules involved in the immune response, including IL-2, CD80 [146], CD40, and CD40 ligand (CD40L) [3]. Additionally, AKNA oversees various processes such as epithelial–mesenchymal transition (EMT) [147], development, neurogenesis, inflammation, autoimmunity, and cancer [142]. Notably, AKNA, acting as a tumour suppressor, serves as a prognostic indicator [142] and influences susceptibility to various cancers, including acute lymphoblastic leukaemia [148], neck squamous cell carcinoma (HNSCC) [149], cervical cancer (CC) [141,144], and gastric cancer (GC) [147]. Part of its influence is attributed to AKNA’s ability to regulate the immune system (reviewed [142]). In the context of cervical cancer, for instance, the E6 protein of high-risk human papillomavirus (HR-HPV) interacts with and downregulates both AKNA and its downstream target CD40, thus compromising immune surveillance [144].

### 2.6. Chromatin Regulator Complexes and AT-Hooks: The BRG1/BRM-Associated Factor (BAF)

A glance at Figure 1 and Supplementary Table S1 reveals that several chromatin-regulating complexes contain AT-hooks. Chromatin structure is extensively remodelled throughout development and cell life. Chromatin remodelling is a dynamic process that enables cells to perform all DNA-related functions, such as duplication, transcription, repair, and recombination, and to respond to various stimuli [150]. This dynamicity is regulated by chromatin factors. Some of them add PTM to histones (writers), while others remove these modifications (erasers), making this process reversible. Additionally, protein complexes, called “readers”, recognise these modifications and enable chromatin remodelling complexes to adjust chromatin compaction by repositioning nucleosomes. Since the discovery of the first histone PTMs, acetylation and methylation of lysine [151], and a large number of histone PTMs, such as propionylation, butyrylation, and finally lactylation, have been discovered and continue to be discovered [152]. In parallel, a large number of protein domains have been identified that can specifically recognise these PTMs. These domains enable the recruitment of factors to chromatin in specific regions where they can add further PTMs on histones and/or remodel chromatin. Recent findings have demonstrated that members of different families of reader domains such as bromodomains, PHD fingers, PWWP, and chromodomains, frequently have the ability to bind DNA. Interestingly, this DNA-binding ability can sometimes occur independently of their histone-binding activity [150]. For several reader domains, the nucleic acid-binding activity has been shown to enhance their affinity for nucleosomes. Furthermore, additional nucleic acid-binding motifs have been described, which work in tandem with the reader domain to strengthen nucleic acid association through supplementary contacts [150]. AT-hooks were found to be present and contribute to DNA-binding due to their ability to contact AT-rich regions in the minor groove.

The BRG1/BRM-associated factor (BAF) complex belongs to the SWI/SNF family of remodellers. The ATPase subunit of BAF can be either BRM or BRG1. These two subunits are mostly divergent in sequence in the N-terminal region, while they are otherwise highly conserved, especially in the C-terminal region, where some auxiliary domains are located (Figure 3). These include an AT-hook and a bromodomain (BD) separated by a short (6 aa) linker. The BD not only binds acetylated histones but can also bind DNA. However, the DNA-binding activity is strengthened by the adjacent AT-hook [153]. Specifically, the AT-hook and BD span the minor and major grooves, respectively, and there is evidence suggesting that they are positioned at the nucleosome interface near the H3 tail and the entry DNA [14,154,155]. The importance of this motif (AT-hook, linker, and BD) for BAF activity is underscored by the high conservation of all three elements across most animal lineages, backed by structural data [153]. This is also supported by the observation that conserved residues within the K/R-rich and RGRP elements of the AT-hook, as well as K/R residues within the BD basic patch, are mutated in cancer [153,156,157]. A recent comprehensive investigation into the role of the AT-hook within the regulatory domain of SWI/SNF demonstrated that it enhances remodelling by modulating the intrinsic DNA-dependent ATPase activity of the catalytic subunit. Remarkably, this effect occurred independently of the AT-hook’s ability to promote SWI/SNF recruitment to DNA or nucleosomes, as no change in substrate affinity was detected. This suggests that the AT-hook serves additional functions beyond its known roles [158]. Moreover, the authors provided further evidence for the evolutionary significance of the AT-hook, highlighting its essential role in the transcriptional activation of stage-specific enhancers critical for cell lineage priming in mouse embryonic stem cells [158].

### 2.7. Mixed Lineage Leukaemia 1 (MLL1)

MLL1 is a lysine-specific methyltransferase (also known as KMT2A and ALL-1) that catalyses the transfer of methyl groups to lysine 4 in Histone 3. It belongs to the MLL family of histone methyltransferases, which comprises six members (MLL1-4, SETD1a, and SETD1B), all of which are characterised by the presence of a SET domain, which is responsible for the enzymatic activity. Three members (MLL1, MLL2, and MLL3) possess a variable number of AT-hooks (two in MLL1, five in MLL2, and only one in MLL3) (Figure 3), which bind AT-rich sequences, inducing chromatin modifications and transcriptional coactivation [159]. In particular, the three MLL AT-hooks interact with regions attached to the nuclear envelope and periphery of the nucleolus and colocalise with topoisomerase II at mitotic chromosomal scaffolds, suggesting a role in regulating chromatin structure [160]. In addition to the AT-hooks, MLL1 contains numerous conserved functional motifs: one menin-binding domain (MBD), two speckled nuclear localisation domains (SNL), four plant homeodomain fingers (PHD), two breakpoint cluster regions (BCR), one bromodomain (BD), two caspase cleavage sites (CS1 and CS2) where the native protein undergo cleavage into two fragments (MLL-N and MLL-C), and two interaction domains (FYRN and FYRC) enabling the association of MLL-N and MLL-C after cleavage. Additionally, it possesses a transactivation domain (TAD) and the SET domain, endowed with H3K4 histone methyltransferase activity, all of which coordinate the multiple functions carried out by MLL1 (Figure 3) [161,162].

MLL1 is highly expressed during early development and haematopoiesis at sites of active transcription and DNA repair. Its rather weak methyltransferase activity requires the presence of additional components, such as WDR5, RBBP5, and ASH2L, for the full activation [163].

Chromosomal translocations, which are associated with aggressive forms of leukaemia, both in adults and children (such as acute lymphoblastic leukaemia—ALL) [161], lead to disruption of the MLL1 gene. This results in fusion proteins containing the N-terminal portion of MLL1, including the AT-hooks, fused with 1 of 80 heterologous partners, thereby replacing the C-terminus [162]. In this case, the AT-hooks are functional for the leukaemogenic process, due to their DNA-binding activity. Accordingly, they are never found mutated in leukaemic patients, regardless of whether they carry MLL1 rearrangements or not [164,165]. Chimeric partners lead to aberrant gene transcription (reviewed in [166]). Recently, a non-canonical function was identified, showing that MLL1 is able to methylate the non-histone target Borealin K143 in the intrinsically disordered region essential for the liquid–liquid phase separation of the chromosome passenger complex (CPC) [167], thereby regulating genome stability. The inhibition of MLL1 activity perturbs CPC phase separation, impairing sister-chromatid cohesion. In hepatocellular carcinomas (HCCs), this leads to growth suppression due to chromosome instability and aneuploidy [167]. 

### 2.8. Methyl-CpG-Binding Protein 2 (MeCP2) 

MeCP2 is a “reader” of methylated CpG islands, where its interaction with other proteins induces heterochromatin clustering [168] and transcriptional activation or repression, thereby regulating cell function, metabolism, and identity [169]. Its high expression in neurons is consistent with its major role in neurological and neurodevelopmental disorders, including X-linked forms of autism spectrum disorders and Rett syndrome. Additionally, recent studies have highlighted its oncogenic role when overexpressed in human cancers [170], including breast, pancreas, liver, and lung carcinomas [169]. In cancer cells, MeCP2 activates the MAPK and PI3K signalling pathways, mimicking the action of RAS in malignancies [170]. 

In addition to its methyl-CpG binding domain (MBD), MeCP2 has three AT-hooks (AT-hook 1, aa 184–195; AT-hook 2, aa 264–273; AT-hook 3, aa 341–364), located in the C-terminal portion of the protein [171], which confer methylation-independent DNA-binding and, seemingly, overall chromatin organisation capabilities (Figure 3) [172]. Mutations in the MBD or AT-hook domains have been identified in autism and Rett syndrome [8,173,174]. Accordingly, the deletion of eight conserved amino acids in the AT-hook 1 domain leads to behavioural and cognitive deficits in mice [174].

Interestingly, a completely new function has recently been attributed to these AT-hook domains. It was shown that they confer low diffusion properties and are responsible for the dynamic behaviour of MeCP2 in granule cells [175]. 

### 2.9. Yeast S. Pombe Orc4 and Epstein–Barr Virus Nuclear Antigen 1 (EBNA1)

DNA replication is a process that must be highly coordinated during the cell cycle so that the DNA molecule is duplicated only once before cell division. Despite differences in the complexity and structural organisation of the genome, the process of DNA replication in all organisms begins with the formation of the pre-replicative complex (preRC) at the so-called replication origins [176,177]. These DNA regions are bound by a multiprotein complex known as the replisome. The origin of replication complex (ORC) is the main component of this macromolecular complex, establishing a structural platform upon which all required factors for DNA duplication—including the helicases for DNA strand opening, the replication fork formation, and the DNA polymerases for synthesising new DNA strands—are coordinated [176,177].

A crucial point is how the ORCs recognise the origins of replication, as this mechanism is different in different organisms. In the budding yeast S. cerevisiae, for example, replication origins are characterised by specific sequences called autonomously replicating sequences (ARS). Conversely, in the fission yeast S. pombe, replication origins are characterised by a high AT content. Notably, one of the ORC components, Orc4, has an N-terminal AT-hook domain involved in the binding of ORC to the DNA replication origin (Figure 3) [176,177,178,179].

In metazoans, the mechanisms by which ORC ends up at the origin of replication are more complicated, as the chromatin landscape can vary greatly across the developmental stages. Recognising the origin of replication within such diversity represents a considerable challenge [176,177]. Several factors could be involved in the specification of replication origins in metazoans, including ORC-interacting proteins, nucleosome positioning, histones PTMs, and other chromatin-related structural features. This complex and dynamic chromatin milieu has prompted the exploration of mechanisms that do not rely on sequence specificity [176,177].

The particular nucleic acid-binding properties of the AT-hook also play a special role in strategies for the formation of preRC in metazoans. In research investigating the Epstein–Barr virus DNA duplication mechanism, the AT-hooks located in the N-terminal region of EBV nuclear antigen 1 (EBNA1) (Figure 3) were identified as essential for the recruitment of the ORC to the Epstein–Barr virus origin of replication OriP [180]. Additionally, these AT-hooks were implicated in ORC recruitment via an RNA-mediated mechanism [16]. Indeed, a G-rich RNA molecule is essential for mediating the interaction between the ORC and EBNA1 [16]. It is also interesting to note that the AT-hooks of HMGA1 can replace the N-terminal AT-hooks of EBNA1 [16]. Apart from facilitating DNA replication through an RNA-mediated mechanism, the AT-hooks have been shown to interact with ORC members both in vitro and in vivo. HMGA1, for instance, can recruit the ORC to AT-rich DNA sequences and colocalise with it in AT-rich heterochromatin. Importantly, this interaction is functional for DNA replication [181]. Taken together, these pieces of evidence suggest a possible role for AT-hooks in replication origin recognition and DNA replication.

### 2.10. AT-Hook-Containing Proteins and Genome Organisation

The DNA targets of AT-hooks are AT-rich DNA sequences, and it is important to note that certain “genomic regions” are particularly rich in AT, namely the scaffold/matrix attachment regions (S/MARS) and the satellite DNA sequences. The former are involved in the attachment of DNA to the nuclear matrix forming topological independent DNA loops, while the latter are tandemly repeated sequences that are highly enriched in centromeric, pericentromeric, and telomeric regions [182,183]. It has been indeed described that AT-hooks are important for localisation to these structures. AT-hook motifs are essential for the binding of stromal antigen 1 (SA1) to telomers [184], for the localisation of the Drosophila D1 protein to centromeric heterochromatin [185], and for the binding of Chromobox 2 (Cbx2) to AT-rich major satellites [186]. The binding of centromere protein A (CENP-A) to the centrosome in yeast is also a mechanism that relies in part on the presence of an AT-hook [187]. The HMGA1 protein is able to bind base-unpairing regions within MARs. Several AT-hook-containing proteins interact with silent AT-rich pericentromeric chromatin (PCH), a peculiar constitutive heterochromatin located on both sides of the centromeres, and contribute to its structural organisation [188]. Intriguingly, what distinguishes some of them, such as Drosophila melanogaster D1 and proliferation disruptor (Prod) as well as mouse HMGA1, is that they mediate the bundling of pericentromeric satellite DNA into ‘chromocenters’, the cytological structures necessary for chromosomes encapsulation into a single nucleus [189,190]. Accordingly, HMGA1 KO phenocopies the presence of defective chromocenters, characterised by micronuclei formation and karyotypic abnormalities [191].

### 2.11. Minor Groove Binders (MGBs) as AT-Hook Competitors

Given their involvement in various cellular processes, including those associated with cancer and other diseases, inhibitors of AT-hook-binding proteins have garnered significant interest. Although the lack of ligand-binding sites has led to the general opinion of their “undruggability”, AT-hooks represent a suitable bull’s-eye: accordingly, molecules able to compete with the AT-rich DNA target sequence, known as minor groove binders (MGBs), have been taken into consideration to block AT-hook-containing proteins and their oncogenic potential. MGBs represent a unique class of molecules that interact with the DNA minor groove and alter the structure and function of the DNA. This mode of action makes them potent inhibitors of various DNA-associated processes, such as transcription, replication, and repair, providing a valuable mechanism for anticancer therapy. Several MGBs have been explored and utilised in clinical settings, showcasing both the therapeutic potential and challenges associated with this approach.

Studies focussing on targeting HMGA proteins have demonstrated that this still growing class of molecules, including netropsin, berenil, dystamicin, Hoechst 33258 (reviewed in [192]), trabectedin [193], sorocein [194], and others, inhibits HMGA1 and HMGA2 binding to target sequences in “cell-free” systems and inhibits cancer hallmarks, such as cell proliferation, migration, and EMT, in cellular and in vivo settings [195,196] as well as diverse physiological and pathological processes, such as inflammation [195] and adipogenesis [197]. While the efficacy of each compound depends on the target expression level [198], unexpectedly, their use can also lead to a reduction of HMGA1 and HMGA2 expression at both the mRNA and protein level [195,196]. This outcome is undoubtedly advantageous, but the underlying molecular mechanism remains still unknown. 

A significant drawback of MGBs is their non-specific binding to DNA. As a result, they target both cancer and normal cells, leading to severe side effects. Accordingly, with the exception of Trabectedin, MGBs are not used clinically but serve as valuable tools in biochemical studies to understand AT-hook protein/DNA interactions and analyse their cellular and molecular impact. Therapeutic compounds that selectively inhibit the binding of oncogenic AT-hook proteins to their DNA recognition sites would be preferable. Suramin belongs to this class of AT-hook inhibitors, as it binds the HMGA AT-hook and not the AT-rich DNA [199]. However, its promiscuity in targeting not only AT-hooks but also unrelated proteins [200] makes it currently too nonspecific, again limiting its clinical application due to potential side effects.

Trabectedin, marketed under the name Yondelis, is a marine-derived compound originally isolated from the sea squirt *Ecteinascidia turbinata*. It has received approval for the treatment of soft tissue sarcoma and relapsed ovarian cancer [201]. However, while its clinical application highlights the potential of MGBs to target and disrupt critical DNA interactions in cancer cells, future research is focussed on improving the specificity of MGBs through structural modifications and combination therapies that selectively target cancer cells while sparing normal tissue. 

Advances in drug delivery systems, such as nanoparticle-based carriers or aptamer-based specific targeting [202], also hold promise for increasing the therapeutic index of these agents. Additionally, understanding the molecular mechanisms underpinning the interactions of MGBs with DNA will guide the design of next-generation binders with optimised efficacy and safety profiles.

## 3. Conclusions

**Figure 4:**
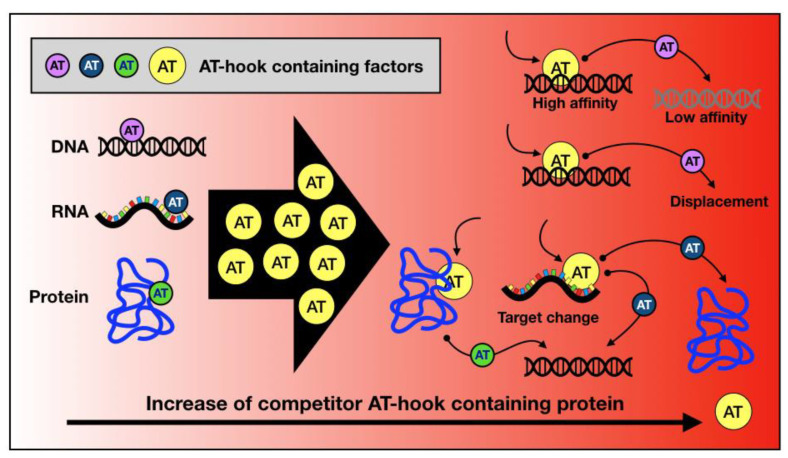
Competition between AT-hook-containing proteins. The AT-hook domain is involved in DNA, RNA, and protein binding. When an AT-hook-containing protein is over expressed, it is conceivable that it could compete with other AT-hook-containing factors. The competition could lead to the occupation of other lower-affinity binding sites, displacing them from targets and potentially changing the type of targets (i.e., DNA -> RNA or PROTEIN; RNA -> DNA or PROTEIN; PROTEIN -> DNA or RNA).

This review shows evidence that there are several factors exploiting the AT-hook domain for their activity. In our opinion, an underestimated aspect regarding AT-hook-containing proteins is the potential for competition between them. Specifically, it has been widely demonstrated that the expression of AT-hook-containing proteins, particularly those belonging to the HMGA family, is strongly increased under certain conditions. These chromatin architecture factors are highly expressed during embryonic development and cell neoplastic transformation, reaching levels just below those of highly abundant chromatin proteins like histone H1. It is plausible that in such a scenario, proteins characterised by the exclusive presence of AT-hooks as DNA-binding domains could displace other AT-hook-containing proteins from the DNA or at least contribute to their redistribution. In addition, many factors possessing AT-hooks also feature other DNA- or chromatin-binding domains [203], and therefore, this could lead to the redistribution of the displaced proteins according to the DNA affinity of the other DNA-binding domain (Figure 4). Indeed, AT-hooks are often located in close proximity to other chromatin/histone reader domains, working synergistically to support the landing of these chromatin/histone readers on DNA. The association of AT-hooks with (i) the bromodomain in BRG1 and (ii) the chromodomain in CBX2 exemplifies how AT-hooks co-operate with other chromatin docking motifs. Could we envision the existence of an “AT-hook network”, wherein subtle changes in the presence of AT-hook-containing factor coordinately impact the functionality of the network? The effect of competition has already been described for paralogous transcription factors and strongly influences genome occupancy and regulatory functions [204].

Another intriguing aspect concerns the binding specificity of different AT-hooks. As shown in Figure 1, in addition to the highly conserved GRP AT-hook core, the N- and C-terminal AT-hook extensions are quite different (albeit retaining some general features). It has been extensively demonstrated that different AT-hooks have distinct preferences for binding to nucleic acids [171]. Furthermore, when multiple AT-hooks are present within the same protein, the spacing of AT-rich DNA sequences strongly influences the overall DNA-binding affinity of AT-hook-containing factors [205]. These findings suggest, therefore, that if a reciprocal functional relationship exists among AT-hook-containing proteins, this could be linked to the AT-rich sequence spacing and distribution.

An additional peculiarity of AT-hook is its “non canonical” ability to interact with other macromolecules, such as RNA. Very recently, it was reported that the AT-hook of SWI/SNF preferentially binds RNA over DNA and associates with the RNA transcribed from enhancers (eRNA) [206]. The authors report data that suggest SWI/SNF is recruited, thanks to the AT-hook interaction, by eRNA to cell-type-specific enhancers in early mammalian development. In this way SWI/SNF facilitates the recruitment of cofactors to stage-specific enhancers and super-enhancers that regulate the transcription of genes involved in cell lineage priming [206]. eRNA are also involved in enhancer/super-enhancer liquid/liquid phase separation processes in conjunction with intrinsically disordered proteins [207]. Many of the AT-hook-containing proteins, including the most representative ones, the HMGA, have intrinsically disordered regions. Therefore, these findings add another level of complexity to the role of AT-hook-containing proteins, which may also be involved in transcription-related processes through non-DNA-mediated mechanisms. eRNA binding and AT-hook involvement in liquid/liquid phase separation processes may well represent a new scientific frontier in terms of the biological functions of AT-hooks.

## Data Availability

Not applicable.

## Supplementary Materials

The following supporting information can be downloaded at https://www.mdpi.com/article/10.3390/ijms25168863/s1.

## Abbreviations

3′-UTR—3′-untranslated region; 5′-UTR—5′-untranslated region; AHL—AT-hook motif nuclear localised; AKNA—AT-hook-containing transcription factor; ALL—such as acute lymphoblastic leukaemia; AP—apurinic/apyrimidinic; ARS—autonomously replicating sequences; ASH2L—absent, small, or homeotic 2-like protein; BAF—BRG1/BRM-associated factor; BAX—Bcl-2–associated X protein; BCC3—basal cell carcinoma, susceptibility to, 3; BCL-2—B-cell lymphoma 2; BCR—breakpoint cluster regions; BD—bromodomain; BER—base excision repair; BRD—bromodomain; BRD4—bromodomain-containing protein; BRG1—Brahma-related gene 1; BRM—Brahma; CaATL1—capsicum annuum AT-hook-like gene 1; CaATL1—capsicum annuum AT-hook-like gene 1; CBX2—Chromobox protein homolog 2; CBX2—Chromobox protein homolog 2; CC—cervical cancer; CC—cervical cancer; CDKN1A—cyclin dependent kinase inhibitor 1A; CDKN1A—cyclin dependent kinase inhibitor 1A; CDKN1B—cyclin dependent kinase inhibitor 1B; CDKN1B—cyclin dependent kinase inhibitor 1B; CENP-A—centromere protein A; CENP-A—centromere protein A; CENP-B—centromere protein B; CENP-B—centromere protein B; ChIP—chromatin immunoprecipitation; ChIP—chromatin immunoprecipitation; ChIP-Seq—chromatin immunoprecipitation DNA sequencing; ChIP-Seq—chromatin immunoprecipitation DNA sequencing; CPC—chromosome passenger complex; CPC—chromosome passenger complex; CR—charged region; CR—charged region; CR—charged region; CR—charged region; CREB—cAMP response element-binding protein; CS—caspase cleavage sites; CS—caspase cleavage sites; CxxC—CxxC domain; CxxC—CxxC domain; DAXX—death domain-associated protein; DBD—DNA-binding domains, DNA—deoxyribonucleic acid; TFs—transcription factors; dRP—deoxyribo-5′-phosphate; eAT-hook—extended AT-hook; EBNA1—Epstein–Barr Virus nuclear antigen 1; EBV—Epstein–Barr virus; EGR1—early-growth response protein 1; EMT—epithelial–mesenchymal transition; FYRC—F/Y-rich C terminus domain; FYRN—F/Y-rich N terminus domain; GA—glycine (G) and alanine (A) repeat region; GC—gastric cancer; GR—glycine (G)- and arginine (R)-rich domain; HCCs—hepatocellular carcinomas; HDGF—hepatoma-derived growth factor; Hi-C—High Chromosome Conformation Capture; HIV-1—human immunodeficiency virus 1; HIV-1 TAR—HIV-1 transactivating response element; HMG—High Mobility Group; HMGA—High Mobility Group A; HNSCC—neck squamous cell carcinoma; Hox—homeobox; HR-HPV—high-risk human papillomavirus; HRP-3—HDGF-related protein 3; HSA—helicase/SANT-associated domain; IBD—integrase-binding domain; IBD—integrase-binding domain; ID—intrinsically disordered; IFN-beta—interferon-beta; IL6—interleukin 6; IMS-MS—Ion-Mobility Spectrometry–Mass Spectrometry; IN—integrase; KAT2B—lysine) acetyltransferase 2B; KLF6—Kruppel-like factor 6; KO—knock out; KOA—knee osteoarthritis; LBD—LEDGF-binding domain; LEDGF/p75—lens epithelium-derived growth factor 75; MAPK—mitogen-activated protein kinase; MAZR—MAZ-related factor; MBD—menin-binding domain; MBD—methyl-CpG-binding domain; MBD—methyl-CpG-binding domain; MBM—menin-binding motif; MDM2—mouse double minute 2 homolog; MeCP2—methyl-CpG-binding protein 2; MeCP2—methyl-CpG-binding protein 2; MGBs—minor groove binders; MLL1—mixed lineage leukemia 1; mRNA—messenger RNA; N-CoR—nuclear receptor co-repressor; ncRNA—non-coding RNA; NLS—nuclear localisation signal; NMR—nuclear magnetic resonance; ORC—origin of replication complex; ORC—origin of replication complex; OriP—origin of plasmid replication; PATZ1—POZ/BTB and AT-hook-containing zinc finger protein 1; PCD—primary ciliary dyskinesia; PCH—pericentromeric chromatin; PCR—polymerase chain reaction; PEST—proline (P), glutamic acid (E), serine (S), and threonine (T) domain; PEST—proline [P]-, glutamic acid [E]-, serine [S]-, and threonine [T]-rich domain; PHD—plant homeodomain; PHD—plant homology domain; PI3K—phosphoinositide 3-kinase; PIC—pre-integration complex; POZ/BTB—POxvirus and Zinc finger/Broad-complex, Tramtrack and Bric a brac; preRC—pre-replicative complex; Prod—proliferation disruptor; Psip1—PC4- and SF2-interacting protein 1; PSR/JMJD6—phosphatidylserine receptor, Jmj-domain-containing protein D6; P-TEFb—positive transcription elongation factor-b; PTMs—post-translational modifications; PWWP—proline (P), tryptophan (W), tryptophan (W), proline (P) domain; QLQ—glutamin (Q), leucin (L), glutamin (Q) motif; RAS—rat sarcoma; RBBP5—retinoblastoma-binding protein; RNA—ribonucleic acid; RNA-Seq—RNA sequencing; RNF4—RING finger protein 4; S/MARS—scaffold/matrix attachment regions; SA1—stromal antigen 1; SELEX—systematic evolution of ligands by exponential enrichment; SET—Drosophila proteins Su(var)3-9, Enhancer-of-zeste and Trithorax domain; SET—Su(var)3-9, Enhancer-of-zeste and Trithorax domain; SMRT—silencing mediator of retinoic acid and thyroid hormone receptor; SnAC—Snf2-ATP coupling domain; SNL—speckled nuclear localisation domains; snRNP—small nuclear ribonucleoprotein; SP1—specificity protein 1; SWI/SNF—SWItch/sucrose Non-Fermentable; TAD—transactivation domain; TAD—transactivation domain; TdIF1—terminal deoxynucleotidyltransferase-interacting factor 1; TGFB1—transforming growth factor beta 1; Tip5—TTF1-interacting protein 5; TRD—transcriptional repression domain; TSS—transcription start site; UR—unique region; WDR5—WD repeat-containing protein 5; Win—WDR5 interaction motif; ZBTB19—zinc finger and BTB protein 19; ZFAT—zinc finger and AT-hook domain containing; ZNF278—zinc finger protein 278.
